# Artificial Intelligence Analysis of Outdoor Sports Risk Self-Assessment on Insurance Psychology

**DOI:** 10.3390/ijerph20043140

**Published:** 2023-02-10

**Authors:** Zhiling Chen, Xinghong Dai, Zhigang Tan

**Affiliations:** 1 School of Physical Education, University of South China, Hengyang 421001, China; 2 School of Physical Education, Hunan University, Changsha 410082, China

**Keywords:** insurance psychology, artificial intelligence analysis, outdoor sports risk, association vector machine

## Abstract

The development potential of China’s medical insurance market is huge, and the research on medical insurance demand has always been the focus of academic discussions. As a result, the discipline of behavioral economics is derived, which aims to explain the decision-making behavior of individual insurance consumption. Among them, the focus of this study was to investigate the influence of individual psychological characteristics and cognitive level on insurance behavior under the difference of reference points. This paper combined behavioral insurance, actuarial mathematics and the econometrics knowledge system, comprehensive theoretical analysis, and empirical tests and analyzed the impact mechanism of individual frame effect on medical insurance demand under different reference points at multiple levels. At the same time, based on the risk self-assessment of outdoor sports, the artificial intelligence of insurance psychology was analyzed. Based on the correlation vector machine algorithm and the theoretical basis combined with the dual perspective of insurance products, the expected utility model was established under the “guarantee framework”, and the prospect theoretical model was established under the “profit and loss framework”. The framing effect was used to measure the relative size of “guarantee utility” and “profit and loss utility”, and a high-insurance-rate model and a low-insurance-rate model were established. The theoretical model analysis found that under the high insurance rate, because the “profit and loss utility” is positive, the size of the individual frame effect is positively correlated with the willingness to insure. Under the low insurance rate, because the “profit and loss utility” is negative, the size of the individual frame effect is negatively correlated with the willingness to insure. The research results of this paper show that insurance is an important beginning of insurance consumption behavior, which includes the complex mentality and emotion of consumers on insurance activities. The insurance demand of policyholders is formed by the joint action of external and internal incentives. Many factors such as income level and education level play an important role in insurance consumption decision making.

## 1. Introduction

In the early 1980s, outdoor sports began to develop in China. Today, outdoor sports have experienced nearly 30 years of development in China [1]. With the development of economy and society, the urban living environment is no longer people’s pursuit. The noisy environment of modern cities, the fast pace of life, and the polluted air have become a burden on people, bringing various urban diseases to people. Outdoor sports emphasize the integration of sports and nature, and it has become a new way of life that closely combines sports, tourism, and culture. Outdoor sports advocate a healthy and happy lifestyle and emphasize self-challenge, teamwork spirit, and outdoor environmental protection so as to pursue the harmony between man and nature. Outdoor sports clubs, which play an important role in sports development, have become a focus of attention. Meanwhile, there is an increase in outdoor sports accidents, in which many people got injured or even lost their lives. For example, according to the “2021 China Outdoor Adventure Accident Report” released by the China Adventure Association, the number of accidents in outdoor adventure activities in China increased by 187 in 2021, with the death toll increasing by 280 percent. These casualties remind people that the safety of outdoor sports cannot be ignored in the process of outdoor sports development, and it is necessary to pay more attention to the prevention of outdoor sports risks while focusing on development. Therefore, the artificial intelligence analysis of outdoor sports risk self-assessment of outdoor clubs on insurance psychology has been studied. The construction of the outdoor risk prevention mechanism of outdoor clubs has a better guarantee for the personal safety of outdoor sports consumers, which is of great significance for the healthy and rapid development of the entire outdoor sports industry.

It is becoming more and more important to use AI technology to study insurance psychology. The field of behavioral economics combines insights from the field of psychology to explain discrepancies between the predictions of traditional economic theory and actually observed behavior. Richter et al. understood and predicted the correlation between customer behavior and the insurance industry and deeply understood the impact of behavioral factors on better evaluation and interpretation of customer behavior [2]. Harikrishnan et al. used statistical methods based on financial time series for seasonal variations and correlations and ultimately generated trading signals for insurance data [3]. Tseng used scenario-based questionnaires for data collection and partial least squares regression for testing. The results showed that respondents’ views on moral strength and fairness were affected by fraud types [4]. After the concept of “machine learning” was put forward, artificial intelligence technology achieved cross-domain and interactive development, which brought great changes and innovations to the traditional insurance industry. Fang found in practice that the effective integration of artificial intelligence and Internet technology realized the intelligent and automatic operation of insurance sales, customer service, underwriting, claims, and other links. He further strengthened the consumption experience of insurance consumers and promoted the improvement of consumer demand, which provided a guarantee for the sustainable development of the insurance industry [5]. Zhuang et al. proposed an auto insurance business analysis method that used three mixed types of data clustering algorithms to segment customers [6]. However, there is still insufficient research on artificial intelligence analysis of insurance psychology.

Appropriate physical activity is important for both physical and mental health. Using an existing mobile app, Fernandez et al. collected self-reported data on daily outdoor activities, emotional well-being, and the impact of the COVID-19 pandemic on participants’ outdoor activity levels between April and July 2020. This work suggested that being outdoors might improve mental health during the COVID-19 pandemic [7]. In view of the physical and psychological characteristics of contemporary college students, Ouyang pointed out through a large number of questionnaires and on-site interviews that outdoor sports had a positive impact on the physical development of college students, which could promote healthy development of their physical fitness and improve their physical function level so as to enhance their ability to adapt to the external environment. Outdoor sports had a positive impact on the psychology of college students, which could develop their minds and abilities and relieve pressure and college students’ bad moods so that they could develop the concept of lifelong exercise. College students’ emotions were developed, and their good motivation for sports hobbies were cultivated. Their willpower to not admit defeat was cultivated, and their healthy personalities were developed [8]. Jackson et al. believed the COVID-19 pandemic challenged human health and well-being. He used the Qualtrics XM team to conduct a national survey of participants 10–18 years old (n = 624) from 30 April to 15 June 2020. In a representative survey, the study found a strong correlation between changes in outdoor activity and changes in subjective well-being [9]. Al-Anouti et al. conducted an online survey of 245 participants to assess the relationship between residents’ daytime and nighttime outdoor activity levels and mental health during the COVID-19 pandemic; the link between nighttime and better mental health may be attributable to vitamin D, but more in-depth research was needed to confirm it [10]. Outdoor play increases the risk of contracting or spreading the coronavirus. The study of Barron and Emmett found the impact of COVID-19 restrictions on children’s and adolescents’ play, their access to and use of outdoor spaces, and their friendship groups [11]. To summarize, most theories are still simple analysis at the macro level. In the past two years, a few scholars have begun to conduct detailed research on outdoor sports risk management, but the related theoretical system is not perfect enough, and further research is needed to improve it. 

## 2. Artificial Intelligence Analysis Method of Outdoor Sports Risk Self-Assessment on Insurance Psychology

### 2.1. Artificial Intelligence and Psychological Prediction

The process of human social cognition is similar to that of the artificial neural network. Therefore, many researchers have established artificial neural network prediction models with different characteristics for some psychological variables in the process of social cognition. Therefore, this paper links artificial intelligence with psychological prediction and constructs relevant models.

The goal of psychological research is to predict and control human behavior and to describe and explain the general laws of psychological phenomena by exploring the explicit behavior of research objects. As an important auxiliary method, AI plays an important role in the measurement and prediction of psychological variables.

More importantly, the combination of big data and artificial intelligence can use environmental behavior data and artificial intelligence technology to realize the automatic identification of human psychological indicators, namely the environmental identification method, which greatly expands the research and application field of psychology.

### 2.2. Risks of Outdoor Sports

Outdoor sports are a group of group projects in nature. Activities include mountaineering, rock climbing, skydiving, camping, picnics, orienteering, expeditions, fishing, and many others [12]. The many outdoor recreational sports are adventurous, extreme, sub-extreme, challenging, and exciting, encouraging one to embrace nature and challenge oneself.

**Figure 1:**
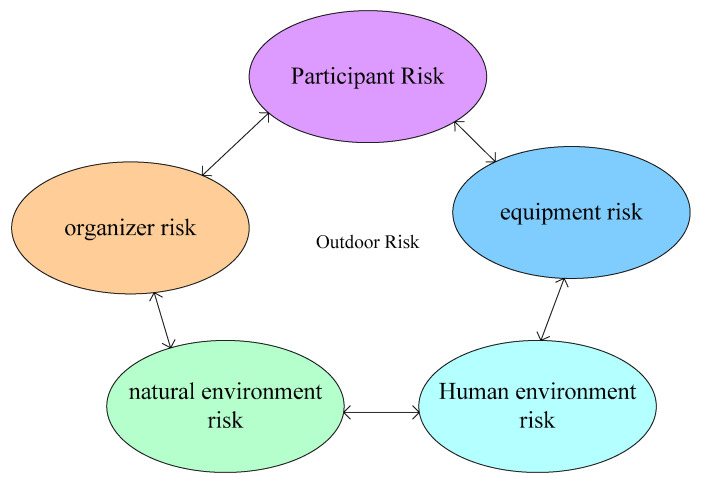
Influencing factors of outdoor sports risks and safety accidents.

Due to the variety of activities and uncertain venues, outdoor sports are challenging and have certain risks, which is exactly the charm of outdoor sports. The influencing factors of outdoor sports risk safety accidents are shown in Figure 1.

As shown in Figure 1, the risks of outdoor sports mainly include environmental risks, and the uncertainty of natural environment and weather may bring risks, personnel risks, the diversity of participants, and the lack of understanding of the activities; and the lack of mastery of relevant technical knowledge may bring risks [13] such as the risk of activity organization, the feasibility, scientificity of the activity plan, the supportability in the implementation process, and equipment risks. Improper selection, use, and management of outdoor sports equipment may bring risks and losses [14]. Outdoor sports often have a wide range of activities, and some social conflicts that may be encountered bring corresponding risks to the activities. The following two main sub-risks, namely participant risk and natural environment risk, are mainly studied.

(1) Participant risk

Participant risk mainly refers to the occurrence of risk accidents caused by factors such as physical health, psychological status, outdoor sports skills and skills mastered, and personal moral quality of outdoor sports participants [15]. It mainly includes risk accidents caused by participants’ excessive physical energy consumption during outdoor sports or due to their own special physical diseases, sudden recurrence of injuries, etc. There are risk accidents caused by factors such as risk taking, luck, distraction, and weak self-protection awareness when participating in outdoor sports activities. When participating in outdoor sports, the participants do not have or are not familiar with outdoor sports technology and can lack emergency and first-aid skills and other factors, which lead to risk accidents.

(2) Natural environment risks

Outdoor sports are carried out in natural venues, and the complex and changeable natural environment is an important factor affecting the risk of outdoor sports. It mainly includes getting lost due to violent storms; inadaptability of human body function caused by continuous high temperature and high cold; risk accidents caused by earthquake, debris flow, mountain torrent, collapse, and rockfall; risk accidents caused by complex terrain such as steep slopes, cliffs, deep streams, marshland, or desert quicksand; and risk accidents caused by fierce animal attacks, snake and insect bites, or toxic and complex vegetation [16].

### 2.3. Psychological Prediction Algorithm Based on Artificial Intelligence

The development of artificial intelligence in the field of psychological prediction is very rapid, and the main algorithm steps are explained below.

First, let the training data set be ${\left\{x_{n},t_{n}\right\}}_{n=1}^{N}$, of which ${\left\{x_{n}\right\}}_{n=1}^{N}$ is the eigenvalue of the training data, and the target vector is $t={\left(t_{1},\ldots ,t_{N}\right)}^{T}$. The following formula is satisfied between the target value and the eigenvalue:

(1) x=t+φ=σw+φ

Among them, *w* is the parameter vector; *φ* = [$\phi_{1},\ldots ,\phi_{M}$] is the *N* × *M* dimension matrix.

To obtain a sparse Bayesian framework, the error term is assumed to follow a Gaussian distribution with mean 0 and variance *σ*2 [17]. The Gaussian likelihood of the target *t* is as follows:

(2) $$p(x|w,\alpha^{2})={\left(2\pi \right)}^{-N/2}\alpha^{-N}\exp\{-\frac{{\left||x-t|\right|}^{2}}{2\alpha^{2}}\}$$

*w* controls the contribution of each prior term to the above formula, with a full-valued normal distribution. According to Bayesian inference, if *a* is known, then

(3) $$p(w|x,\beta ,\alpha^{2})=p(x|w,\alpha^{2})p(w|x,\beta )/p(w|\beta ,\alpha^{2})$$

Gaussian distribution *N*(*μ*, 2), A is a diagonal matrix; in order to estimate the model parameter *w*, the maximum value of the marginal likelihood distribution $p(w|\beta ,\alpha^{2})$ is first solved, which is equivalent to solving the maximum value of its logarithm *L*(*a*):

(4) $$L(\beta )=\log p(x|\beta ,\alpha^{2})=\log\int_{-\infty }^{+\infty }p(x|w,\alpha^{2})p(w|\beta )dw\;=\frac{1}{2}[N\log2\pi +\log|C|+x^{T}C^{-1}x]$$

(2) Classification principle of association vector machine

The solution of the association vector machine classification problem is carried out under the framework of the regression problem. The difference is that the target value in the classification problem is assumed to obey the Bayou distribution, and the Sigmoid function is introduced, that is,

(5) $$p(x_{n}|w)=\alpha \{t{\left(x_{{}_{n}};w\right)}^{x_{n}}\}[1-\alpha {\left\{x_{{}_{n}};w\right)}^{{1-x}_{n}}]$$

Obviously, it is easy to know that neither the posterior probability *p*(*w*|*x*,*a*) nor the marginal likelihood distribution *p*(*x*|*a*) can be obtained in the analytical form through the integral form [18].

For the current fixed value *a*, the solution of $W_{MP}$ is equivalent to the solution of the maximum of the following formula:

(6) $$\log(p(x|w)p(w|\beta ))=\sum_{n=1}^{N}\left[x_{n}\log t_{n}+(1-x_{n})\log(1-t_{n})\right]-\frac{1}{2}w^{T}Aw$$

The Laplace method is a quadratic estimation algorithm for the log posterior distribution. The second derivative of the above formula is as follows:

(7) $$\nabla_{w}\nabla_{w}\log p{\left(w|x,\beta \right)}_{w_{MP}}=-(\Phi^{T}B\Phi +A)$$

Through the algorithms and steps, artificial intelligence technology can be used to predict human mental health more quickly and accurately. This algorithm has made a huge contribution in the field of psychological prediction.

### 2.4. General Law of Consumption Behavior and Its Performance in Insurance Consumption

(1) Pursuing utility maximization

Although the pursuit of the principle of utility maximization has been criticized by many researchers, especially experts in the fields of evolutionary economics and behavioral economics, it has to be admitted that the pursuit of the principle of utility maximization still has directional guiding significance. The view that consumers do not adjust their consumption mix means that their marginal utility in various consumptions is equal or that the total utility is the largest, which also has certain problems. For various other reasons, even if consumers know that other consumption combinations are better, that they cannot choose the situation is an important challenge to this understanding.

The duration of the pursuit of utility maximization has been defined differently in different theories [19]. Before the emergence of life cycle theory, economists mostly defined such a period from short term and long term. In fact, consumers’ life-time arrangements are indeed based on income and expenditure expectations. However, some may be conservative: if one’s expected income is less, and the expected expenditure is more, the resulting financial arrangements may result in the formation of redundant legacy. Others may be aggressive, with optimistic expectations for income and conservative expectations for spending. As a result, the financial arrangement may form a negative legacy. Others consider the expenditure of their next generation more. In this way, their financial arrangements are based on the family as a unit and take the duration of at least two generations to consider their utility variables.

(2) Following the habitual principle

The habitual principle means that the current behavior of consumers is influenced by past habits. If people are habitually not using insurance to solve the so-called risk problem, it is indeed difficult to change this habit. However, young and middle-aged consumers are theoretically more receptive to using insurance to deal with risk issues. Similarly, relatively speaking, people in the more open and faster-developing eastern regions may be more likely to change the traditional risk-transfer mechanism and accept the new risk-management mechanism of insurance.

(3) Irreversibility

The change of people’s consumption level is relatively slow; in other words, once a certain consumption level is formed, it is difficult for consumers to change, whether in terms of motivation or living habits [20]. As the old Chinese saying goes, “It is easy to turn from frugality into luxury, and it is difficult to turn from luxury to frugality”. This can serve as an important justification for insurance consumption, especially for those consumers motivated to save. They are worried that there will not be as much income in the future to protect current consumption (which is more serious if inflation is taken into account). At the same time, people’s motivation for consumption of security products also contains the consideration of how to ensure the current consumption level in the face of the uncertainty of future income.

(4) Exemplary

In the “relationship group” formed by a certain social relationship, the consumption patterns of the members within the group have a strong demonstration role among the members; at this time, it can also be called the “reference group” of consumption [21]. In addition, when people’s values emphasize social identity rather than individuality, the exemplary role in consumer behavior is more prominent. Chinese traditional culture emphasizes social identity more. Therefore, the demonstration role of consumers is also more obvious. It is manifested in the insurance practice that people tend to buy insurance products purchased by their “reference group” and tend to choose insurance companies and insurance agents that more people choose.

(5) Dynamic development

With the advancement of science and technology, the development of economy, and the prosperity of culture, people’s consumption is constantly changing. The object, structure, level, and quality of consumption are in the process of constant change, update, and optimization. This dynamic change of consumption behavior has infinite characteristics. This means that even if insurance consumption is not the current choice of a representative consumer, it may be his future choice. The current characteristics of insurance consumers may change their attitudes towards insurance consumption with the development of social economy and changes in their own attributes (such as increasing income level, increasing age, getting married, and having children, etc.) [22].

## 3. Artificial Intelligence Experiment of Outdoor Sports Risk Self-Assessment on Insurance Psychology

The fluctuating development trend of new payment in separate accounts of investment-linked insurance reflects the important role of individual financial knowledge and risk awareness in influencing insurance demand. It should be pointed out that the “profit and loss utility” of medical insurance is not equivalent to the return of medical insurance risk investment. The former is to examine the utility of medical insurance products in the “profit and loss framework”, and the risk probability is the probability of an accident occurring within the scope of medical insurance. The latter is an investment attribute attached to medical insurance, and the risk probability is based on investment market fluctuations. In order to comprehensively consider the mechanism of framing effect on China’s medical insurance demand, it is critical and necessary to explore the influence of “profit and loss utility” on medical insurance demand. Chowdhury, Subrata developed an effective machine learning system integrated with the Internet of Things to predict the health insurance amount. He believed that the Internet of Things in healthcare enabled interoperability, machine-to-machine communication, information exchange, and data movement, thus making healthcare service delivery effective. In addition, the work simulation system he developed to solve the task of medical insurance cost prediction showed significantly improved accuracy [23].

### 3.1. Variable Selection

In order to explore the impact of individual framing effect on medical insurance demand, the empirical model established in this paper uses the medical insurance demand index as the explained variable and insurance “protection utility” and individual frame effect surrogate variables as explanatory variables. In order to ensure the goodness of fit of the model, some control variables are selected in combination with related research.

Based on these analyses, this paper mainly divides the empirical variables into three categories. The first is the demand for medical insurance, which includes statistics on social basic medical insurance and commercial medical insurance. The second is the substitution variable of the individual frame effect, including directly measuring the size of the frame with the proportion of risky assets and indirectly measuring the size of the frame effect with individual cognitive differences (memory and computing power) and regret factors (experiences in danger). The third is other control variables, including measuring the level of individual wealth and other characteristics. The variable descriptive statistics are shown in Table 1.

As shown in Table 1, from the perspective of insurance participation, the sample participation rates of social basic medical insurance and commercial medical insurance are 92.6% and 3.14%, respectively. Obviously, there are differences in the willingness of individuals to purchase the two types of medical insurance. This difference is the basis for the later analysis of the impact mechanism of the frame effect based on the reference point difference. Considering the influence of the income effect of insurance demand, this paper takes the logarithmic regression of the value of individual total assets. The average value of individual total assets is about 78,000, of which risk assets account for about 2.22%. It can be seen that the overall sample’s recognition of risk is not high. The average education level is 4.29: the average level of just graduating from primary school and not reaching the level of junior high school graduation. The average age of the individual is 59 years old. From the perspective of individual cognitive ability, the individual cognitive ability is good, and the subtraction and memory tests of 7 are all above 3 points. The average medical experience of the individual in the past year is 0.24.

### 3.2. Model Construction

According to the previous theory, the individual frame effect is a negative effect at a low insurance rate; that is, the higher the individual frame effect, the stronger the individual’s willingness to insure. The individual frame effect is a positive effect at high insurance rates; that is, the lower the individual frame effect, the stronger the individual’s willingness to insure. Based on this, this paper models the bottom insurance rate and the high insurance rate separately and illustrates the differential impact of the individual framing effect through different medical insurance data.

**Figure 2:**
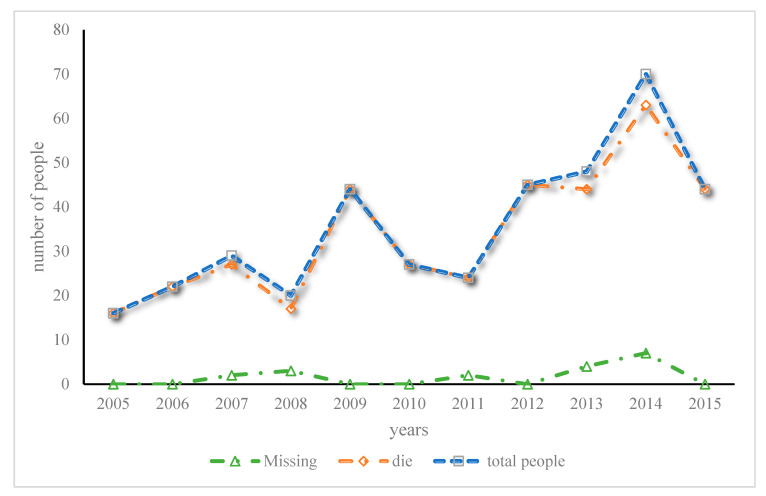
Trends in the number of casualties in mountaineering and outdoor sports from 2005 to 2015.

This paper takes typical and related mountaineering accidents as examples, aiming to better understand and analyze the characteristics and causes of outdoor accidents in China. Therefore, this paper takes the outdoor sports accidents that have occurred in China in recent years as an example to analyze the current situation and characteristics of outdoor sports accidents in China. Figure 2 shows the changing trend of the number of victims of mountaineering outdoor sports from 2005 to 2015.

**Figure 3:**
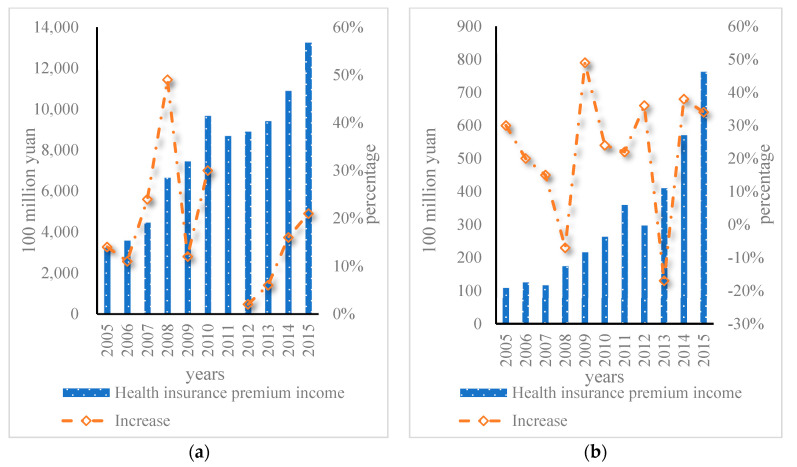
Commercial health insurance premium income, 2005–2018. (**a**) Health insurance premium income and increase. (**b**) Health insurance claims and benefits and increase.

As shown in Figure 2, according to the latest statistics on participation in outdoor sports, the number of people participating in outdoor sports in China reached 130 million in 2015, of which about 60 million people participated in mountaineering, hiking, and crossing. The premium income of commercial health insurance from 2005 to 2018 is shown in Figure 3.

**Figure 4:**
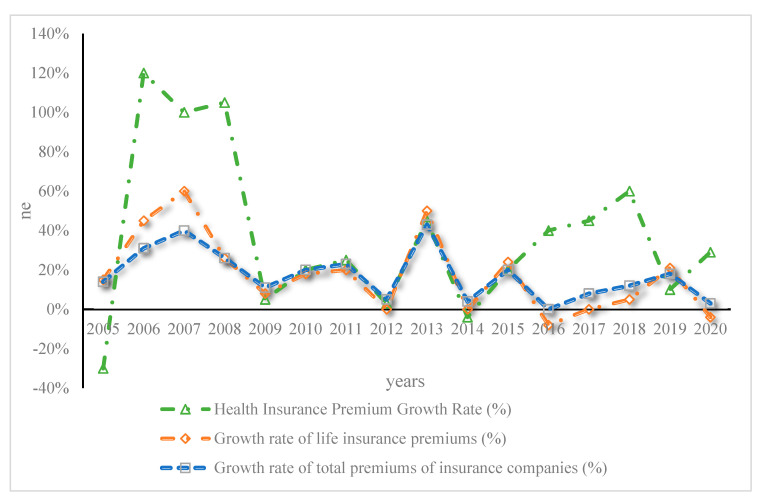
Comparison of the growth rates of the three types of premium income from 2005 to 2018.

As shown in Figure 3, the medical insurance premium was only CNY 2.8 billion in 2005, while the premium income in 2018 was CNY 487.91 billion, with an average annual growth rate of 33.75%, and the growth rate was rapid. Figure 4 shows the comparison of the growth rates of the three types of premium income from 2005 to 2018.

As shown in Figure 4, compared with the average growth rate of insurance companies’ total premium income from 2005 to 2018, the average growth rate was 19.49%, and the average annual growth rate of life insurance premiums of insurance companies was 20.18%. It shows that the average annual growth rate of medical insurance premiums in China is much higher than the average annual growth rate of total premiums and life insurance premiums.

### 3.3. Deconstruction of Outdoor Sports Medical Insurance Results

Insurance products have the basic function of insurance protection [24]. Under the “guarantee framework”, the most basic motivation of consumers’ demand for medical insurance products comes from the psychology of individuals seeking safety and stability. The role of health insurance is to provide compensation to the insured after hospitalization due to illness to ensure recovery from treatment. This also illustrates the fundamental essence of insurance with security. However, under the background of the continuous development and innovation of medical insurance, the development of social basic medical insurance is also faced with many restrictive factors.

**Figure 5:**
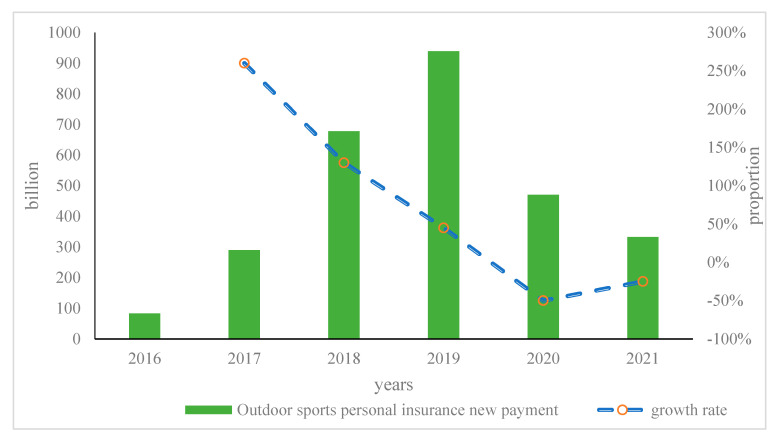
New payment and growth rate of outdoor sports personal insurance.

Under the “profit and loss framework”, medical insurance is non-immediate, risk accidents are based on future predictions, and the occurrence of risk accidents is uncertain, so insurance benefits are also uncertain. That is to say, under the “profit and loss framework”, medical insurance itself is a risk and can be understood as an option with additional conditions. This option awareness and risk perception depend on certain financial knowledge. Families with a higher level of financial literacy generally have a better understanding of the nature of insurance risks and thus have a stronger insurance awareness. Under the “profit and loss framework”, the incentive for individuals to insure comes from risk–reward. Therefore, an insurance product with a clear risk–reward value is more likely to be classified by the policyholder under the “profit andloss framework”. One example is the development of investment-linked insurance, which has a partially self-inflicted investment risk-gain/loss nature. The new payment and growth rate of outdoor sports personal insurance is shown in Figure 5.

As shown in Figure 5, to a certain extent, people can see the market demand of China’s investment-linked insurance. The data show that in 2017, the new payment for China’s outdoor sports personal insurance was CNY 28.95 billion, with a growth rate of 248.06% in that year. During 2019, it achieved continuous growth, but its growth rate showed a downward trend, and it showed a negative growth trend in 2017. This showed that during the epidemic period from 2019 to 2021, outdoor sports personal insurance continued to decline, proving the impact of the epidemic on outdoor sports insurance. The development of outdoor sports personal insurance turned from prosperity to decline, which reflects that rational policyholders would not increase their insurance demand simply because of the additional risk investment function on the basis of insurance product protection functions. The improvement of insurance would make the choice of insurance products more “cautious”. It can use sample data to analyze the impact of individual framing effects on insurance purchase behavior under different reference points. Table 2 shows the regression results of the model with high and low coverage rates.

As shown in Table 2, it can be seen that the range of goodness of fit is between 0.04 and 0.05, which means that it is significant at the 95% significance level, and the modified score data are authentic. The high-coverage-rate model and the low-coverage-rate model are integrated, in which the high-coverage-rate model describes the social basic medical insurance market, and the low-coverage-rate model describes the commercial medical insurance market. Under different market entities, in order to avoid the autocorrelation between different explanatory variables from affecting the regression results, this section establishes regression models for different explanatory variables. In order to ensure the robustness of the model, this section adds social medical insurance as a commercial medical. The control variable of the insurance demand model, on the one hand, explores the impact of social medical insurance demand on commercial health insurance demand. On the other hand, it verifies whether the explanatory power of the explanatory variable personal frame effect on commercial health insurance demand changes after adding new control variables. Table 3 shows the regression results of the commercial medical insurance demand model after adding social medical insurance coverage.

It can be seen from the table that the fitting results are less than 0.05, which indicates that the fitting results of this paper are excellent. At the same time, the interaction coefficient between social medical insurance and medical experience is not significant. It is proven that under the empirical data of this paper, the substitution effect or promotion effect of social medical insurance on commercial medical insurance is not obvious, or the two effects may cancel each other out. Therefore, it is reasonable to build a model without considering the impact of social medical insurance on commercial medical insurance before.

### 3.4. Deconstruction of the Psychology and Behavior of Insurance Consumers

Consumers have motivation to consume because of their needs, and when they have motivation, they understand the object to be consumed through cognition and learning. These cognitions and understandings form an attitude towards the intended consumption object, and these attitudes may also change, and so on. Obviously, it is necessary and feasible to introduce the general law of consumer behavior into the research of insurance consumption behavior.

**Figure 6:**
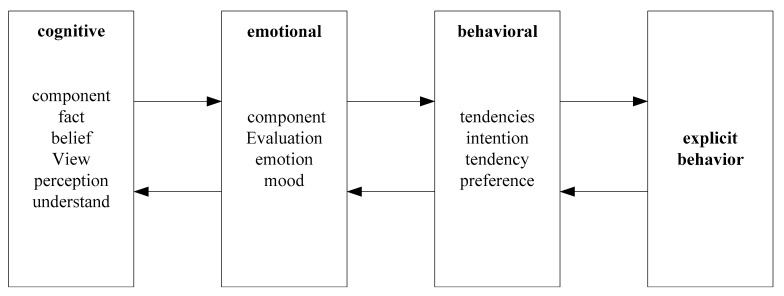
Attitude components and their relationships.

Obviously, attitudes have different reasons, but once an attitude is formed, it has a key impact on consumers’ insurance consumption decisions. In order to further expand the insurance market and tap the potential of insurance consumption, it is necessary to change the attitudes of consumers who are indifferent, rejecting, and hostile towards insurance. The components of attitude and their relationships are shown in Figure 6.

As shown in Figure 6, in general economic analysis of insurance consumption, researchers often strip away the personality factors of decision makers. They do not need to know a series of personalized factors such as the age, gender, ethnicity, income level, occupation, education level, etc., of the decision maker. However, an indisputable fact is that consumers often show completely different consumption characteristics because of the above factors rather than showing the only choice for utility maximization under the so-called income constraint. At the same time, insurance consumption activities must follow the general rules of human psychology and behavior, so it is necessary to introduce the theory of consumption psychology and behavior to the analysis.

### 3.5. Process and Impact of Insurance Consumer Decision Making

(1) Rational decision-making process of insurance consumers

**Figure 7:**
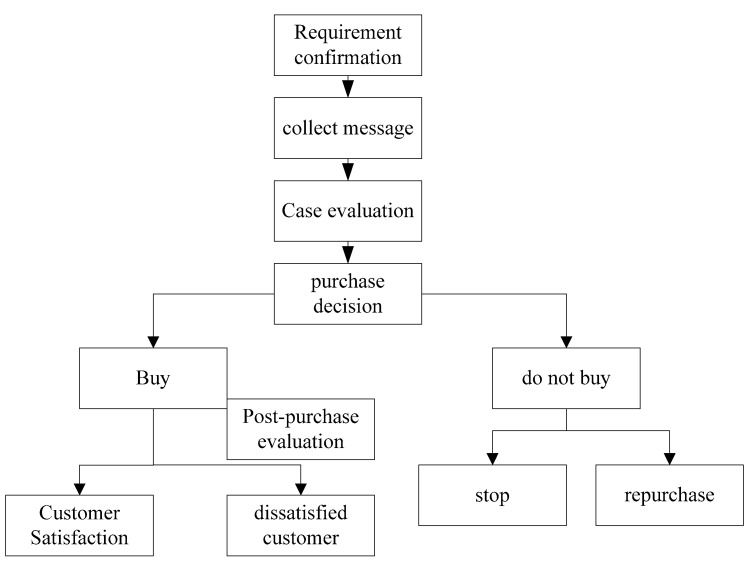
Schematic figure of insurance consumer spending.

What factors might motivate people to buy insurance? First of all, consumers’ personal risk is a continuous state. Therefore, the so-called driving factor should be to stimulate consumers to find their own risks and achieve a certain degree of risk management. Secondly, the confirmation of demand also requires consumers to reach a certain level of income. The schematic figure of insurance consumer consumption is shown in Figure 7.

As shown in Figure 7, the insurance demand of policyholders is formed by the combined action of external and internal stimuli. For example, when consumers worry that they may lose their labor force and lose their source of income when they are old, this demand for maintaining a future life prompts them to have a demand for insurance, and this demand is completed under the internal stimulus. When consumers see accidental death in outdoor sports, they realize the idea of purchasing personal accident insurance for outdoor sports. This demand is completed under the stimulation of the outside world.

(2) Insurance psychology and behavior

Insurance is an intangible commodity. Strictly speaking, insurance commodities should include financial services such as risk management. However, in the personal insurance consumer market, what consumers mainly receive is a contract from an insurance company. The contract represents a responsibility and a promise. The insurance company promises to give a certain amount of insurance compensation or payment within a certain period of time and under certain conditions, which can be understood as a conditional option. This right is non-immediate, taking a long time to obtain, or even not occurring at all. Insurance is an important beginning of insurance consumption behavior, which includes the complex mentality and emotion of consumers towards insurance activities.

## 4. Conclusions

Insurance psychology refers to people’s subjective feelings such as approval or hesitation, affirmation, or negation of insurance when they participate in or will participate in actual insurance activities. It includes the perception, perception, thinking, emotion, willingness, and other cognitive activities of insurance. The psychological process of insurance includes the motivation process and behavior process. The motivation to seek security is universal, but insurance behavior is not universal. Taking the Chinese medical insurance market as an example, this paper conducts an empirical study of information asymmetry and discusses the existence and possible manifestations of information asymmetry in the Chinese medical insurance market, which will help to design more targeted policies and improve market quality. The making of purchase decisions is also closely related to consumers’ own personality and self-concept. Therefore, the researchers of insurance marketing theory also studied various strategies to promote consumers to make purchase decisions according to the characteristics of consumers’ different personalities. In addition, making purchase decisions for insurance consumption seems to be more difficult than many other consumption activities. The uncertainty of the expected occurrence of future insurance accidents and the uncertainty of whether the insurance company can fulfill the obligations advocated by the insurance marketing personnel constitute the main psychological obstacles for consumers to make decisions. At the same time, consumers are faced with the control and intervention of consumption behavior in many future periods when purchasing multi-year, regular payment insurance, which is also the reason why consumers find it difficult to make these decisions. 

## Figures and Tables

**Table 1:** Variable descriptive statistics.

Variable	Mean	Standard Deviation	Minimum	Maximum Value
gender	0.547293	0.497789	0	1
age	59.48504	9.433346	20.8333	102.5
mar	0.859634	0.347387	0	1
edu	4.290662	1.7738	0	10
logassets	11.2682	1.710257	4.605 I 7	23.4316
R/T	0.022244	0.093455	0	0.972179
sub7	4.077147	1.161387	0	5
mem	3.292116	1.999013	0	10
treatm	0.244883	0.430044	0	1

**Table 2:** Model regression results of high and low insurance rate models.

Variable	High-Insurance-Coverage Model			Low-Insurance-Rate Model		
gender	0.0839 *	0.0811 *	0.099 **	0.0145	0.0324	0.0208
age	−0.0023	−0.0017	−0.0036	−0.0188 ***	−0.0169 ***	−0.0192 ***
mar	0.016	0.0057	0.0188	0.2235 **	0.2099 **	0.2242 **
edu	0.0299 **	0.0163	0.03 **	0.0786 ***	0.0578 ***	0.0807 ***
logassets	−0.0198 *	−0.0215 *	−0.0167	0.0703 ***	0.0694 ***	0.0732 ***
R/T	−0.7328 **	-	-	−0.5614 ***	0.0394	−0.0479
Constant	1.6127 ***	-	1.6047 ***	−2.177 ***	−2.5166 ***	0.0523
goodness of fit	0.044	0.045	0.041	0.042	0.048	0.044

Note: ***, ** and * indicate statistically significant at the 1%, 5%, and 10% level, respectively.

**Table 3:** Regression results of social medical insurance and commercial medical insurance models.

Variable	Low-Insurance-Rate Model	Variable	Low-Insurance-Rate Model
age	−0.0186 ***	R/T	−0.5742 **
gender	0.0142	treatm	0.1971
mar	0.2241 **	S_ ins	−0.0427
edu	0.0791 ***	Treatm*s_ ins	−0.2640
logassets	0.0235 ***	Constant	−2.1335 ***
goodness of fit	0.036	0.035	0.032

Note: ***, ** and * indicate statistically significant at the 1%, 5%, and 10% level, respectively.

## Data Availability

The data that support the research findings are available on request. The data are not publicly available due to the privacy of research participants.
